# Effects of a family home-based intervention on global health and 24h movements: an exploratory family cluster-based approach to childhood obesity

**DOI:** 10.3389/ijph.2026.1609165

**Published:** 2026-06-25

**Authors:** Julie Siroux, Juliette Lemay, Inès Ramos, Vicky Drapeau, Jean-Phillipe Chaput, Elodie Vedrine, Carla Dalmais, Victoria Rousset-Thiry, David Thivel, Yves Boirie, Magalie Miolanne

**Affiliations:** 1 Clermont Auvergne University, EA 3533, Laboratory of the Metabolic Adaptations to Exercise Under Physiological and Pathological Conditions (AME2P), CRNH Auvergne, Clermont-Ferrand, France; 2 Specialized Center for Obesity in Auvergne (CSO CALORIS), CHU Gabriel Montpied, Clermont-Ferrand, France; 3 The Faculty of Agriculture and Food Sciences, School of Nutrition, Laval University, Québec, QC, Canada; 4 Unit of Biostatistics (DRCI), Clermont-Ferrand University Hospital, Clermont-Ferrand, France; 5 Department of Kinesiology, Faculty of Educational Sciences, Laval University, Québec, QC, Canada; 6 Department of Pediatrics, Faculty of Medicine, University of Ottawa, Ottawa, ON, Canada; 7 International Research Chair Health in Motion, Clermont Auvergne University Foundation, Clermont-Ferrand, France; 8 Department of Human Nutrition, CHU Gabriel Montpied, Clermont-Ferrand, France

**Keywords:** clusters analysis, home and family-based intervention, movement behaviour, paediatric obesity, perceived health

## Abstract

**Objectives:**

This study aimed at (i) exploring the effects of a 12-month home-based family intervention on perceived global health and 24 h movement patterns among children with overweight/obesity (OW/OB) and their family members; (ii) identifying intra-family behavioral clusters and their influence on the intervention’s effectiveness.

**Methods:**

142 families (n = 223 legal guardians) with at least one child with OW/OB were included. At baseline and 12 months, perceived health, sleep, physical activity (PA) and sedentary behaviors (SB) were assessed.

**Results:**

Children with OW/OB showed poorer physical and mental quality of life (QoL) than normal weight (NW) ones (p < 0.001), and decreased body mass index (BMI) z-score over time (p = 0.001). Guardians with OW/OB improved physical QoL at the end of the program (p = 0.002). For all outcomes, clusters analysis suggested a distinction between NW children and those with OW/OB. Children tended to resemble their guardians of same weight status (WS). Changes in BMI z-scores among children with OW/OB might vary by QoL and behavioral profiles with greatest reductions in clusters having higher baseline global health or PA.

**Conclusion:**

This study suggests the potential of family-centered strategies addressing childhood obesity.

## Introduction

After decades of increasing prevalence, pediatric obesity is now a major threat to future population health. Reports from the World Health Organization (WHO) highlight that over 390 million youth aged 5–19 years were overweight (OW) in 2022, including 160 million living with obesity (OB) [1]. A systematic review and meta-analysis found that 55% of children, and 80% of adolescents with OB will remain affected later in life [2], highlighting the importance of early intervention. Evidence suggests that the factors contributing to childhood obesity often originate at the family level, and that improvements in family lifestyle can positively influence healthy behaviors, helping to reduce the prevalence of childhood obesity [3]. Previous studies suggested an intergenerational transmission of health-related behaviors, with parenting patterns persisting across generations [4–6]. These findings emphasize that childhood obesity is not an isolated issue affecting the child alone but part of a familial context. For example, higher levels of parental physical activity (PA) are associated with increased PA and reduced sedentary behaviors (SB) in children [7], with mothers playing a key role in helping their children meet the 24-h movement guidelines [8]. Similarly, guardians’ sleep habits and overall health-related quality of life (HRQoL) are associated with their children’s sleep quality and duration [9], as well as with perceived health [10].

Multiple settings and approaches have been proposed for the prevention and management of pediatric obesity, ranging from clinics and schools to surgery and pharmacology. Among these, multidisciplinary behavioral interventions remain crucial for reinforcing and sustaining the benefits achieved [11]. As recently demonstrated, adding behavioral components such as family therapy to diet and PA interventions can positively impact children’s body mass index (BMI) and BMI z-scores [11]. Parenting practices promoting healthy behaviors may also be key elements of preventive interventions aimed at reducing the risk of childhood obesity [12]. Given the importance of healthy behaviors in both preventing and managing—and the critical role legal guardians play in shaping their children’s lifestyles—family-based interventions involving not only the child but also their guardians and siblings have become increasingly common. These interventions have shown promise in supporting weight management [13] and improving health behaviors [14]. Often implemented successfully in community or care centers [15], family interventions coordinate nutrition, PA, and psychosocial strategies to improve outcomes for children with obesity. However, long-term studies remain rare, and although many interventions adopt family-centered approaches, few incorporate a home-based component that reflects the family’s everyday life. Additionally, little is known about the impact of these interventions on family members’ health behaviors, and whether distinct intra-family behavioral profiles may influence program success. In this context, the objective is to evaluate the effects of a 12-month family and home-based intervention on perceived health, HRQoL, and 24 h movement patterns (PA, SB, and sleep) among children with OW/OB and their family members. Secondly, the study aims to identify intra-family behavioral clusters and examine their influence on interventions’ effectiveness.

## Methods

### Design

The ProxOb program is a family- and home-based intervention addressing childhood obesity. It engages the family unit and relies on an interdisciplinary team, leveraging parental involvement as a key driver for sustainable lifestyle changes. The program provides tailored advice on PA, SB, nutrition, sleep, psychological and family dynamics. Each family works with a dedicated team of three professionals. Detailed descriptions of the program are available elsewhere [16]. The ProxOb program follows five steps to deliver a personalized intervention: (i) initial assessment: a first meeting with the family, the three professionals (a PA educator, dietician, psychologist, or social worker), the pediatrician, and the program coordinator; (ii) intervention (1 year): a family- and home-based intervention comprising 18 support sessions over 12 months, including three educational assessments, and fifteen workshops (five on nutrition, parenting, and PA); (iii) post intervention assessment: a progress review after the intervention; (iv) maintenance phase (second year): continued support through follow-up calls and online workshops for families; and (iv) long-term assessment: concluding meeting to evaluate the program’s impact, with referrals to additional health services if needed for ongoing support. Our study focused on step 1 to 3 with collected data from February 2021 to November 2024 for all family members.

### Participants

Families were included through a broad, community-based approach across several French departments (Puy-de-Dôme, Allier, Cantal, Haute-Loire, and Drôme–Ardèche). Inclusion was open, with families entering the program via referrals from healthcare providers or on their own. To participate in the program, families must have at least one child under 18 living with OW/OB, defined as a BMI exceeding the International Obesity Task Force (IOTF) threshold of 25. Additionally, they must meet at least one of the following criteria: (i) be classified as socially vulnerable based on the EPICES score (Évaluation de la Précarité et des Inégalités de santé dans les Centres d’Examens de Santé) [17]; (ii) live in a medically underserved area; (iii) lack access to healthcare services; (iv) have previously failed an obesity intervention. Eligible families were also required to demonstrate sufficient availability to complete the program’s questionnaires. The study was conducted in accordance with the Declaration of Helsinki and approved by the Ethics Committee of the Sud Est VI (2016/CE62). The program was also registered with the Commission National de l’Informatique et des Libertés: 1989437V1. All adult participants completed a written informed consent form, and a legal guardian signed the form for youth under the age of 18.

### Outcomes

All family members (guardians and children, being NW or OW/OB) were evaluated, with the measurement process thoroughly detailed in a previous publication [16]. The following section outlines the specific outcomes used in the study.

#### Anthropometry

Body height and weight were measured using a standing stadiometer and a body weight scale (Seca Les Mureaux, France) for all participants. In children, BMI, BMI z-scores and percentiles were calculated, with obesity defined using the IOTF age- and sex-specific BMI curves [18]. For adults, BMI was calculated using the formula weight/height^2^ (kg/m^2^), and weight status (WS) was determined based on WHO reference values [19].

#### Questionnaires

Adults and children (aged 8–17 years) completed questionnaires covering a range of behaviors and overall health at baseline and after the first year of ProxOb intervention. The study design is presented in Supplementary Material 1.

### Health-related quality of life

In adults, Short Form Questionnaire (SF-36), a valid and reliable scale [20] was used to assess HRQoL. It consists of 36 items assessing eight dimensions (physical functioning, physical role, bodily pain, general health, vitality, social functioning, emotions, and mental health), with two final scores (physical and psychological) comprised between 0 (severe dependence) and 100 (independence). For children, HRQoL was assessed using the Pediatric Quality of Life Inventory (PEDSQL), a valid questionnaire designed for individuals aged 2–18 years [21]. It consists of 23 questions, divided into four dimensions (physical, emotional, social, and academic) and scored on a 5-point Likert scale ranging from 0 (never) to 4 (almost always) with age-appropriated versions. After recoding, a physical and psychological health scores between 0 and 100 were obtained (scoring method is available in Supplementary Material 2), with higher results indicating higher HRQoL.

### Perceived health status

The 3-Levels European Quality of Life 5 Dimensions questionnaire (EQ5D-3L), shown to exhibit excellent psychometric properties in people with NW and with OW/OB was used to assess health status in adults [22]. Is based on five dimensions (mobility, autonomy, usual activities, pain/discomfort, and anxiety/depression), each with three levels (no problems, some problems, and extreme problems). Participants rated their health status by selecting the statement that best described their condition. Scores ranged from 1 to 3, with the mean score calculated across dimensions. Higher scores indicated poorer health status. In children over 8 years old, health status was assessed using the 3-Levels European Quality of Life 5 Dimension for Youth (EQ5D-3L-Y), an adapted version of the EQ5D-3L. It contains the same five dimensions formulated in an appropriate way for children (mobility, taking care of myself, doing my everyday activities, pain/discomfort, worried/sad/unhappy), with the same scoring method. This questionnaire has been validated and shown to be reliable and feasible for use in young people with health disorders. Its reliability has also been confirmed in a systematic review of 40 studies, in which the mean age of participants was 11.8 years [23].

### Sleep

To assess sleep duration, participants were asked “*Usually, how much sleep do you get per night during the week?”* and “*Usually, how much sleep do you get per night at the weekend or on holiday?.*” The average of the two responses was then calculated to assign a score between 0 and 3 to sleep duration, with higher sleep duration equating to higher score. For adults, the classification was: < 5 h = 0, 5–7 h = 1, 7–9 h = 2 and > 9 h = 3. For children, the categories were as follows: <7 h = 0, 7–9 h = 1, 9–11 h = 2, and > 11 h = 3. This classification was made according to the sleep duration recommendations for both pediatric [24] and adult populations [25].

### Physical activity and sedentary behaviors

To obtain indicators of PA and SB, adults were asked to complete the Physical Activity Questionnaire of the National Observatory on Physical Activity and Sedentarity (ONAPS-PAQ), a 21-question questionnaire in three parts, covering work, transport, and leisure/home activities during a usual week [26]. This reliable and valid questionnaire in adults aged 18–69 years [26] gives a score for the level of PA and the amount of sedentary time, with higher scores indicating better PA level and higher sedentarity. Children aged 8 years or older filled in the Children and Adolescents Physical Activity and Sedentarity Questionnaire (CAPAS-Q), which enables a similar assessment [27]. This questionnaire has also been validated in children and adolescents aged 8–18 years [27]. Using different contexts, it also provides two scores (PA and SB). The scoring method and interpretation are available in Supplementary Material 2.

### Statistical analyses

Statistical analyses were performed using Stata software (version 15; StataCorp, College Station, TX, USA). All tests were two-sided, with an alpha level set at 5%. Continuous data were presented with mean ± standard deviation and categorical data with number and associated proportions. Longitudinal analyses were performed using mixed linear regression models to assess the effect of the intervention (T0 *vs*. T12), WS (NW *vs*. OW/OB) and sex (female *vs*. male) as fixed covariates, and considering individuals and family as random effects, on health status, sleep, PA and SB. These models were conducted on legal guardians and children separately. Interaction between time and WS was assessed in a complementary model. A Sidak’s type I error correction was applied for multiple pairwise comparisons.

A sensitivity (complete-case) and attrition analysis comparing baseline characteristics between participants with and without T12 data were performed. Model assumptions were checked using residual diagnostics, including assessment of normality and homoscedasticity, and no major violations were observed. Mixed models were estimated using maximum likelihood, allowing inclusion of participants with incomplete data under a missing-at-random assumption. Spearman’s correlations were used to measure the association between BMI z-score change and changes in health status, sleep, PA and SB (Δvalue = value T12 – value T0). Correlation coefficients were interpreted as follows (absolute coefficient value): ≥ 0.70 (strong), 0.40 to 0.69 (moderate), 0.10 to 0.39 (weak), < 0.10 (negligible). To classify participants by their initial characteristics and behavior at baseline, a principal component analysis (PCA) was performed in children, using FactoMineR and Factoextra packages. A multiple component analysis was also performed, categorizing the scores using quartile 1, median and quartile 3 as cut-offs, for children and legal guardians separately. These categories allowed to consider children and adults on the same classification. Hierarchical clustering on principal components was then performed. Cluster analyses were performed using complete case and are considered exploratory. Finally, BMI z-scores were compared between T0 and T12 in each cluster using a paired Wilcoxon test.

## Results

One hundred forty-two families were included in the analysis, representing 477 participants (135 female guardians, 88 male guardians, 60 NW children, and 194 children with OW/OB). Regarding the WS of legal guardians, 79% (n = 107) of females and 85% (n = 75) of males were living with OW/OB (Supplementary Material 3). Sex parity was almost observed in children with OW/OB (with 53% of females and 47% of males) and in NW children (50% of both females and males) All the descriptive characteristics are shown in Supplementary Material 3. The attrition analysis comparing baseline characteristics between completers and non-completers only showed a significant age difference for children (p = 0.022), with no difference observed for adults (Supplementary Materials 4,5).

### Effects of the ProxOb program at the individual level depending on weight status

Tables 1, 2 display all the detailed results for children (Table 1) and adults (Table 2) regarding the effects of the ProxOb program on HRQoL, PA, SB, and sleep at the individual level. Regarding children, results for BMI z-scores, physical and psychological HRQoL, and perceived health status were significantly different between children with OW/OB and their NW counterparts who shown lower BMI z-scores, higher HRQoL and perceived health status (all p-values <0.001) (Table 1), as also found by clusters analyses (Supplementary Materials 6, 7). A significant time × WS interaction was also found for BMI z-score, with the *post hoc* analysis showing significant decrease from T0 (2.5 ± 0.8) to T12 (2.3 ± 0.9) among children with OW/OB (p = 0.006). A significant time effect in BMI z-score was observed in children (p = 0.044), with a corresponding increase in NW children (β = 0.236, 95% CI [0.010, 0.470]) (effect sizes are presented in Supplementary Material 8). Finally, as shown in Supplementary Material 9, no significant correlation was observed between the delta changes in each behavior and the delta BMI z-score of children with OW/OB, between baseline and 1 year of intervention. Specifically, correlation deltas ranged from 0.146 (Δ health status) to −0.500 (Δ PA) and p-values from 0.132 (Δ physical health) to 0.957 (Δ SB).

**TABLE 1:** Evolution of body mass index z-score, health-related quality of life, health status and movement behaviors (sleep, physical activity, sedentary behaviors) in children (Auvergne, France, 2025).

​	All children			NW children		Children with OW/OB		Mixed Models effects NW vs. OW/OB		
​	T0 n = 254	T12 n = 73	*Time*	T0 n = 60	T12 n = 16	T0 n = 194	T12 n = 57	*Time*	*Weight status*	*Time x WS*
BMI z-score IOTF^1^	2.1 ± 1.1	2.1 ± 1.0	0.074	0.6 ± 0.7	0.9 ± 0.3	2.5 ± 0.8	2.3 ± 0.9	**0.044**	**<0.001**	**0.001** ^a^
Physical health (HRQoL)	79.8 ± 13.5	80.2 ± 14.6	0.548	86.8 ± 13.1	83.9 ± 4.2	78.0 ± 13.1	79.4 ± 16.0	0.615	**<0.001**	0.422
Psychological health (HRQoL)	73.8 ± 14.5	73.5 ± 11.7	0.815	82.3 ± 12.4	74.7 ± 8.0	71.8 ± 14.3	73.2 ± 12.4	0.293	**<0.001**	0.191
Health status	1.3 ± 0.3	1.3 ± 0.3	0.300	1.1 ± 0.2	1.2 ± 0.2	1.3 ± 0.3	1.3 ± 0.3	0.680	**<0.001**	0.350
Sleep duration (hrs)	9.5 ± 1.0	9.5 ± 1.0	0.167	9.3 ± 1.0	9.7 ± 0.9	9.5 ± 1.0	9.4 ± 1.0	0.852	0.460	0.387
Sleep duration ^2^ ^,^ ^3^										
0	2 (2)	0 (0)	​	1 (4)	0 (0)	1 (1)	0 (0)	​	​	​
1	28 (25)	11 (28)	​	7 (29)	1 (12)	21 (24)	10 (32)	​	​	​
2	77 (68)	25 (64)	​	15 (63)	6 (76)	62 (70)	19 (61)	​	​	​
3	6 (5)	3 (8)	​	1 (4)	1 (12)	5 (5)	2 (7)	​	​	​
Physical activity level	2.2 ± 0.4	2.2 ± 0.4	0.739	2.2 ± 0.3	2.2 ± 0.5	2.2 ± 0.4	2.1 ± 0.3	0.226	0.942	0.249
Sedentary behaviors level	2.6 ± 0.7	2.7 ± 0.8	0.086	2.6 ± 0.7	2.6 ± 0.9	2.6 ± 0.7	2.7 ± 0.8	0.633	0.930	0.704

Abbreviations.BMI: body mass index, IOTF: international obesity task force, HRQoL: Health-Related Quality of Life, hrs: hours, OB: obesity, OW: overweight, T0: baseline, T12: after 1-year intervention, WS: weight status.

Sex effect was observed for sedentary behaviors, Sex effect was observed for sedentary behaviors (p = 0.008), data not shown for children, contrary to legal guardians for which sex differences are well known and of interest. P-values in bold are < 0.05.

1 Quantitative variables: Mean ± SD, with significant p-value <0.05.

2 Sleep duration divided into 4 categories, as described in Methods.

3 Qualitative variables: n (% in the subsample).

a Significant difference between children with OW/OB, at T0 and children with OW/OB, at T12 (p = 0.006).

**TABLE 2:** Evolution of body mass index, health-related quality of life, health status and movement behaviors (sleep, physical activity, sedentary behaviors) in legal guardians (Auvergne, France, 2025).

​	All legal guardians			Female legal guardians				Male legal guardians				Mixed models effects females *vs.* males			
​				NW		OW/OB		NW		OW/OB		Sex	*Time*	*WS*	*Time x WS*
​	*T0* n = 223	*T12* n = 99	*Time*	*T0* n = 28	*T12* n = 11	*T0* n = 107	*T12* n = 57	*T0* n = 13	*T12* n = 4	*T0* n = 75	*T12* n = 27				
BMI (kg/m^2^) ^1^	32.8 ± 8.1	32.2 ± 5.5	0.902	22.6 ± 1.6	24.5 ± 3.7	35.9 ± 8.1	32.9 ± 5.1	23.7 ± 1.2	25.2 ± 0.6	33.8 ± 5.9	34.1 ± 4.4	0.071	**0.033**	**<0.001**	**0.017** ^a^
Physical health (HRQoL)	71.3 ± 17.8	78.3 ± 17.8	**<0.001**	84.8 ± 8.4	80.3 ± 12.6	66.7 ± 17.1	75.3 ± 16.3	86.3 ± 3.8	86.7 ± 5.5	72.1 ± 19.3	82.0 ± 22.5	0.079	0.252	**<0.001**	**0.002** ^b^
Psychological health (HRQoL)	60.1 ± 18.5	63.4 ± 19.9	0.081	64.0 ± 13.1	61.5 ± 18.7	56.4 ± 18.7	61.4 ± 19.6	70.9 ± 15.0	66.1 ± 10.0	62.4 ± 19.3	68.2 ± 22.0	**0.008**	0.506	**0.035**	**0.110**
Health status	1.4 ± 0.3	1.4 ± 0.3	**0.021**	1.2 ± 0.1	1.3 ± 0.2	1.4 ± 0.3	1.4 ± 0.3	1.2 ± 0.1	1.2 ± 0.2	1.4 ± 0.3	1.3 ± 0.3	0.126	0.683	**<0.001**	**0.571**
Sleep duration (hrs)	8.0 ± 0.8	8.1 ± 1.0	0.115	8.1 ± 0.9	8.1 ± 1.0	8.1 ± 0.9	8.3 ± 1.1	7.8 ± 0.7	7.3 ± 0.4	7.8 ± 0.8	7.9 ± 0.8	**0.002**	0.894	0.627	0.438
Sleep duration ^2,^ ^3^	​	​	​	​	​	​	​	​	​	​	​	​	​	​	​
Very low	0 (0)	0 (0)	​	0 (0)	0 (0)	0 (0)	0 (0)	0 (0)	0 (0)	0 (0)	0 (0)	​	​	​	​
Low	13 (10)	7 (10)	​	2 (13)	1 (12)	4 (6)	4 (9)	2 (22)	0 (0)	5 (12)	2 (11)	​	​	​	​
Moderate	94 (72)	45 (64)	​	8 (53)	5 (63)	48 (74)	25 (60)	6 (67)	2 (100)	31 (76)	13 (72)	​	​	​	​
High	24 (18)	18 (26)	​	5 (34)	2 (25)	13 (20)	13 (31)	1 (11)	0 (0)	5 (12)	3 (17)	​	​	​	​
Physical activity (METs.min^-1^.week^-1^)	96.0 ± 150.9	88.1 ± 115.7	0.753	80.9 ± 92.3	50.1 ± 59.2	77.0 ± 135.6	65.2 ± 91.3	294.9 ± 427.7	221.4 ± 282.0	101.1 ± 108.4	134.9 ± 140.7	**0.016**	0.570	0.109	0.404
Sedentary time (hrs)	7.6 ± 6.3	7.6 ± 4.8	0.755	7.3 ± 5.5	10.1 ± 7.8	8.0 ± 7.5	7.3 ± 3.6	5.8 ± 4.9	4.3 ± 2.3	7.5 ± 4.8	7.7 ± 5.4	0.538	0.194	0.386	0.191

Abbreviations. BMI: body mass index, HRQoL: Health-Related Quality of life, hrs: hours, NW: normal weight, OB: obesity, OW: overweight, T0: baseline, T12: after 1-year intervention, WS: weight status.

1 Quantitative variables: Mean ± SD, with significant p-value <0.05. 2 Sleep duration divided into 4 categories, as the described in Methods. P-values in bold are < 0.05.

3 Qualitative variables: n (% in the subsample).

^a^ No significant differences in *post hoc* analysis.

^b^ Significant difference between guardians with OW/OB, at T0 and guardians with OW/OB, at T12 (p < 0.001).

In adults, results showed significantly higher BMI (p < 0.001), and lower health status (p < 0.001), physical (p < 0.001) and psychological (p = 0.035) HRQoL in individuals with OW/OB compared to NW. Sex differences were observed for psychological health (p = 0.008), sleep duration (p = 0.002), and PA (p = 0.016) with females reporting longer sleep and males having a higher perceived psychological health and PA level. Regarding physical HRQoL, a time × WS interaction was observed (p = 0.002), with improved perceived physical health in guardians with OW/OB at T12 (females: 75.3 ± 16.3; males: 82.0 ± 22.5) compared to baseline (66.7 ± 17.1; 72.1 ± 19.3; p < 0.001) (Table 2). As in children, a significant time effect on BMI was observed in guardians (p = 0.033), with a corresponding increase (β = 1.400, 95% CI [0.110, 2.700]) (all effect sizes presented in Supplementary Material 10).

Importantly, sensitivity analyses yielded similar results for BMI and physical health in guardians, while for children, results were only similar for BMI z-score IOTF, which showed significant time, WS, and interaction effects. Details are provided in Supplementary Material 11 (children) and Supplementary Material 12 (guardians), and data availability in Supplementary Material 13.

### Baseline associations between family members

**FIGURE 1:**
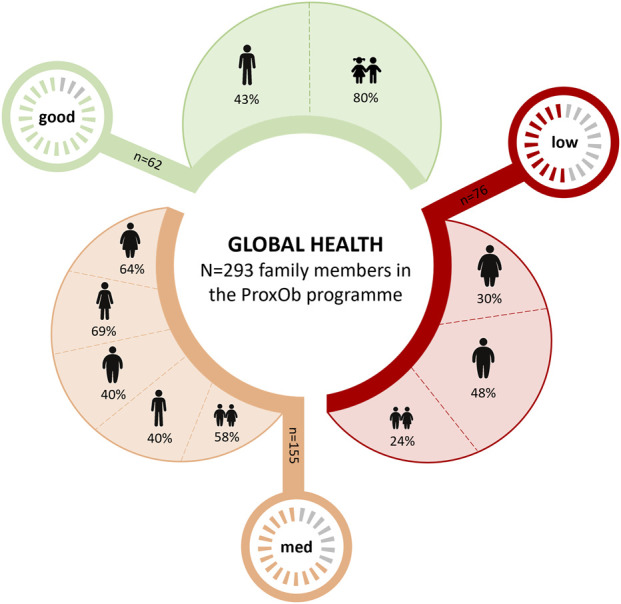
Schematic representation and distribution of family members across three health-related quality of life and health status profiles identified by multi-component cluster analysis (Auvergne, France, 2025). Three distinct family profiles of global health have been identified: the “good global health” cluster (in green) with the best health indicators and comprising mainly children and males legal guardians with normal-weight; the “low global health” cluster (in red) characterized by a high prevalence of overweight/obesity among children and adults; the “moderate global health” cluster (in orange), which is the most common and exhibits intermediate and heterogeneous profiles.

The graphical results of multi-component analysis provide a visual representation of the patterns and associations with families for HRQoL (Figure 1). When considering all family members, the three clusters of HRQoL were widely different, as shown in Table 3, with cluster 3 indicating a better health. This cluster included almost all of the NW children (80%), while those with OW/OB were mostly in clusters 1 and 2, indicating poorer HRQoL. In general, children with OW/OB were close to their legal guardians of same WS, with cluster 1 including almost only guardians and children with OW/OB. Cluster 2 tended to be more heterogeneous, as it also comprised most individuals with OW/OB and almost all NW female legal guardians (69%), suggesting that most females had higher HRQoL than their male counterparts, despite low sample sizes.

**TABLE 3:** Identification of three distinct family clusters based on health-related quality of life and health status using a multi-component analysis: distribution of family members by sex and weight status (Auvergne, France, 2025).

​	Cluster 1 – Low global health (vulnerable profile) n = 76	Cluster 2 – Moderate global health (intermediate profile) n = 155	Cluster 3 – High global health (favourable profile) n = 62
Physical health (HRQoL) ^1^			
Low physical health	68 (90)	20 (13)	0 (0)
Moderate physical health	7 (9)	117 (75)	11 (18)
High physical health	1 (1)	18 (12)	51 (82)
Psychological health (HRQoL)			
Low psychological health	54 (71)	28 (18)	0 (0)
Moderate psychological health	20 (26)	110 (71)	15 (24)
High psychological health	2 (3)	17 (11)	47 (76)
Health status			
High health status	2 (3)	13 (8)	49 (79)
Moderate health status	18 (24)	134 (87)	13 (21)
Low health status	56 (74)	8 (5)	0 (0)
Sex and weight status			
Female legal guardians NW	0 (0)	11 (7)	5 (8)
Female legal guardians OW/OB	23 (30)	49 (32)	4 (7)
Male legal guardians NW	0 (0)	4 (2)	3 (5)
Male legal guardians OW/OB	20 (26)	17 (11)	5 (8)
Children NW	3 (4)	3 (2)	23 (37)
Children OW/OB	30 (40)	71 (46)	22 (35)
Δ BMI z-score, children with OW/OB ^2^	−0.2 ± 0.4	−0.1 ± 0.2*	−0.5 ± 0.7

Abbreviations. BMI: body mass index, HRQoL: Health-Related Quality of life, NW: normal weight, OB: obesity, OW: overweight, Δ BMI z-score: z-score change from baseline (T0) to the end of the intervention (T12).

1 n (% in the cluster).

2 Mean ± SD.

* p-value between T0 and T12 < 0.05.

3 category classification based on a quartile analysis of the scores as follows: low: ≤ Q1, moderate: > Q1 and < Q3, high: ≥ Q3. The cut-offs used for adults are: physical health Q1 (low) = 63.2/Q3 (high) = 87.7, psychological health Q1 (low) = 47.1/Q3 (high) = 75.7, and health status Q1 (high) = 1.2/Q3 (low) = 1.6. For children the cut-off values are: physical health Q1 (low) = 68.8/Q3 (high) = 90.6, psychological health Q1 (low) = 65.0/Q3 (high) = 83.3, health status Q1 (high) = 1.0/Q3 (low) = 1.4.

Although clustering was similar for sleep, PA, and SB, NW female legal guardians and children were all grouped in cluster 3, whereas adults and children with OW/OB were mainly in cluster 1 (Table 4).

**TABLE 4:** Identification of three distinct family clusters based on sleep duration, physical activity and sedentary behaviors using a multi-component analysis: distribution of family members by sex and weight status (Auvergne, France, 2025).

​	Cluster 1 – Sedentary & inactive profile (unhealthy behaviors) n = 105	Cluster 2 – Active profile (healthy behaviors) n = 37	Cluster 3 – Intermediate risk profile n = 26
Sleep duration ^1^			
Low sleep duration	29 (28)	6 (16)	9 (35)
Moderate sleep duration	43 (41)	23 (62)	13 (50)
High sleep duration	33 (31)	8 (22)	4 (15)
Physical activity			
Low physical activity level	35 (33)	0 (0)	5 (19)
Moderate physical activity level	69 (66)	6 (16)	13 (50)
High physical activity level	1 (1)	31 (84)	8 (31)
Sedentary behaviors			
Low sedentary behaviors	28 (27)	10 (27)	6 (23)
Moderate sedentary behaviors	46 (44)	21 (57)	13 (50)
High sedentary behaviors	31 (29)	6 (16)	7 (27)
Sex and weight status			
Female legal guardians NW	0 (0)	0 (0)	11 (42)
Female legal guardians OW/OB	40 (38)	5 (13)	0 (0)
Male legal guardians NW	0 (0)	5 (13)	0 (0)
Male legal guardians OW/OB	18 (17)	15 (41)	0 (0)
Children NW	0 (0)	0 (0)	15 (58)
Children OW/OB	47 (45)	12 (32)	0 (0)
Δ BMI z-score children OW/OB ^2^	−0.1 ± 0.4	−0.4 ± 0.7	-

Abbreviations. BMI: body mass index, NW: normal weight, OB: obesity, OW: overweight, Δ BMI z-score: z-score change from baseline (T0) to the end of the intervention (T12).

1 n (% in the cluster).

2 Mean ± SD.

3 category classification based on a quartile analysis of the scores as follows: low: ≤ Q1, moderate: > Q1 and < Q3, high: ≥ Q3. The cut-offs used for adults are: sleep duration Q1 (low) = 7.5/Q3 (high) = 8.8, physical activity Q1 (low) = 9.3/Q3 (high) = 129.0, and sedentary behaviors Q1 (low) = 3.3/Q3 (high) = 9.8. For children the cut-off values are: sleep duration Q1 (low) = 8.9/Q3 (high) = 10.3, physical activity Q1 (low) = 1.9/Q3 (high) = 2.4, sedentary behaviors Q1 (low) = 2.2/Q3 (high) = 3.2.

**FIGURE 2:**
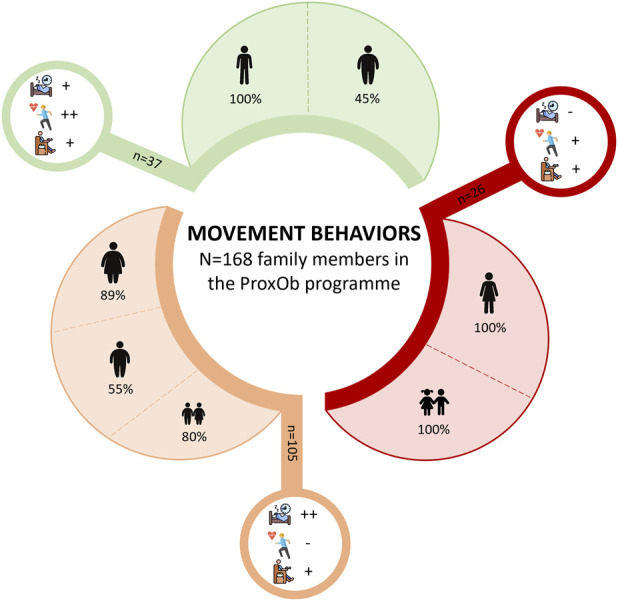
Schematic representation and distribution of family members across three health-related quality of life and health status profiles identified by multi-component cluster analysis (Auvergne, France, 2025). Despite similar sedentary behaviors, three distinct family profiles have been identified, with sleep duration, physical activity level, weight status and sex primarily driving clustering. The better cluster (in green) was male-dominated with only male legal guardians while the lower cluster (in red) included all females legal guardians and all children with normal-weight. The intermediate cluster (in orange), showing low physical activity but high sleep duration included almost all members with overweight/obesity.

As shown in Figure 2, children generally clustered with legal guardians of same WS (Figure 2). Finally, in contrast to clusters 1 and 3, cluster 2 included more males, with higher PA level, and lower SB.

### Baseline behaviors profile and success of the intervention

Changes in BMI z-scores among children with OW/OB between baseline and end of intervention are presented by cluster in Supplementary Materials 6, 7 (children) and Tables 3, 4 (all family members). In children clusters, a significant decrease in BMI z-scores was observed in cluster 2 for HRQoL (−0.3 ± 0.4 [-1.4, 0.3], p = 0.003) (Supplementary Material 6) and in cluster 1 for 24-h movement (−0.3 ± 0.4 [-1.4, 0.5], p = 0.026) (Supplementary Material 7). In family clusters, a significant decrease in BMI z-scores was observed in cluster 2 for HRQoL and health status (−0.1 ± 0.2, p = 0.014) (Table 3) while no change was found for sleep, PA, and SB (Table 4).

## Discussion

Although various interventions exist for childhood obesity prevention and management, incorporating behavioral components and involving the whole family appear key to their effectiveness and sustainability [4, 11]. Leveraging the ProxOb program, this study examined the effects of a home and family-based intervention on HRQoL and movement behaviors (PA, SB, and sleep). These effects were analyzed at the individual family-member level, and an exploratory cluster analysis was conducted to explore intra-family associations. This study also investigated whether baseline associations among family members might be related to the intervention’ success. The findings support the potential effectiveness of the ProxOb program in reducing BMI z-scores in children with OW/OB, although no significant time effects were observed for individual behaviors in mixed-model. Clusters exploratory analyses also revealed distinct patterns of HRQoL and movement behaviors, with similarities in HRQoL and perceived health status across weight categories. Specifically, better physical and psychological health perceptions was associated with improved health status in NW participants. In terms of movement behaviors, higher levels of PA seem to be associated with lower SB, while sleep did not emerge as a key factor in clustering.

Firstly, the observed changes in BMI z-scores align with a previous systematic review and meta-analysis showing that 73% of home-based interventions positively impacted BMI z-scores [28], with reductions up to 37% across 22 home-based interventions [28]. The individual level mixed-model analysis also indicates lower HRQoL in children with OW/OB compared with NW peers, a result consistent with the findings of Van de Pas and colleagues [29]. Consistent with such findings, family-based obesity prevention programs may be among the most effective strategies for targeting childhood obesity and reducing BMI z-scores [30]. Despite differences in WS, our results do not show any effect of the intervention on children’s HRQoL or health status. This lack of intervention effect may partly be explained by the high scores at baseline, a caveat to consider when interpreting the results, as already outlined in literature [31]. Among adults, an effect was observed for physical HRQoL, that significantly improved in guardians with OW/OB post-intervention. This result is in line with a previously conducted 6-month family intervention, with an improvement of the guardians’ general health perceptions [32], although the questionnaire administered was different from the SF-36 used in the present study. Interestingly, this observed improvement occurred in a cohort of adults with a mean baseline BMI of 35.6 kg/m^-2^, highlighting the potential cumulative effects of family obesity interventions on guardians with OW/OB [32].

Regarding movement behaviors, none was modified in response to the ProxOb intervention in children and adults. Interestingly, these findings align with the conclusions of a systematic and meta-analytic review, highlighting that family-based childhood obesity interventions have limited effects on children’s and guardians’ movement behaviors [33]. These results may be explained by practical barriers such as time constraints, competing priorities or unrealistic expectations [34]. Resources limitation and limited access to supportive environments may also have contributed, particularly given the program’s inclusion of socially vulnerable and rural family [35]. Finally, in line with recent studies, the interplay of family dynamics, including cohesion [36], intergenerational influences [37] and adaptive capacity [37], may play a key role in the success of family-based interventions, although these factors were not assessed in the present study.

Interestingly, exploratory cluster analyses showed concordance between legal guardians and children of same WS regarding HRQoL and movement behaviors, in line with prior evidence linking guardian to child health [10] and PA [38]. The intergenerational transmission of obesity and obesity-related behaviors are mediated by genetic and environmental factors, with guardians playing a key role in shaping children’s behaviors [39]. Importantly, this clustering between family members appeared to be higher between children and their mothers, consistent with previous literature highlighting that mothers may have a significant influence on their children’s health and wellbeing [40], but also on PA and SB [41, 42].

Furthermore, as shown by the WS effects analyses of the linear mixed models, our exploratory cluster analysis among children suggested differences by WS, with most NW children being in the same cluster and reporting better perceived HRQoL and health status than their counterparts with OW/OB, in line with previous research showing lower HRQoL scores in children with OW/OB compared to their NW peers [43, 44]. Children with OW/OB also showed reduced psychological health, self-esteem, and physical functioning [44], with such effects observed in both clinical and community-based samples [43], highlighting the importance of considering HRQoL dimensions in obesity prevention and management interventions, as excess weight affects not only biological health, but also overall HRQoL.

With regard to movement behaviors, children were found divided according to their WS, but the results were more heterogeneous, with no difference between children with NW and those with OW/OB. However, studies using subjective questionnaires suggest that children, regardless of WS, report similar sleep duration, PA levels, and SB [45]. This highlights the limitations of self-reported measures, which may partly explain the present findings. In all family member clusters, the observation that higher PA levels may be linked with lower sedentarity highlights the importance of the interplay between PA practice and the reduction of SB.

Finally, although no significant correlations were found between individual health behaviors and changes in BMI z-scores, the exploratory cluster analysis indicated greater reductions in BMI z-scores among children in clusters characterized by better perceived health and higher PA levels. These findings suggest that positive health perceptions and regular PA may serve as key levers for intervention success, potentially enhancing participant engagement and overall effectiveness. This aligns with previous studies showing that children who were already physically active before an intervention experienced the greatest benefits in terms of weight reduction [46]. These results also highlight the complexity of obesity and the fact that others behavioral changes (e.g., eating habits) may be associated with BMI z-scores changes and intervention success. To our knowledge, no prior study has reported a similar association between baseline HRQoL and reductions in BMI z-scores.

All of the present findings should be interpreted in light of certain limitations. These include the subjective nature of measuring PA, SB, and sleep duration—despite the use of validated questionnaires. Additionally, the high rate of missing data limited the scope of the analyses, making it impossible to conduct principal component or multi-component analyses and thereby restricting the ability to interpret the results in cluster form. This also limits the robustness of clustering-based interpretation given the sample size and missing data constraints. The relatively high attrition rate and thus reduced sample size at follow-up, particularly in subgroup and cluster analyses, may also limit statistical power and robustness of these findings. As such, cluster analyses should be considered exploratory and interpreted with caution. Importantly, this attrition rate includes both families who left the program for personal and/or organizational reasons and families for whom data are missing (who did not leave the clinical program) but did not complete the assessments. This should prompt us to reflect on this type of intervention, considering both the organizational aspects and the type of assessment implemented, which should not be too burdensome for families. It would also have been valuable to assess participants’ readiness to change, as this has been shown in other studies to influence intervention outcomes and could have provided further insights. Similarly, family functioning and the context of psychosocial complexity were not analyzed in the present study. With larger sample sizes, it might be interesting to carry out analyses according to the age group of the children and the extent of their excess weight. Importantly, it should be noted that while the ProxOb program offers valuable, innovative, and real-world insights, it was not originally designed as a scientific study. Instead, it is a field-based intervention aimed at preventing and treating childhood obesity at the family level. Although this limits the strength of the conclusions that can be drawn, it also highlights the practical feasibility of implementing large-scale, family-based, home-centered programs—providing unique evidence grounded in real-world practice.

### Conclusion

By integrating behavioral and multidisciplinary approaches, the ProxOb program suggests associations between the participation in the intervention and reduced BMI z-scores in children with OW/OB, as well as increased physical HRQoL in adults with OW/OB. The present study also indicates the potential existence of distinct behavioral phenotypes related to HRQoL, PA, SB, and sleep within family members. These findings reinforce the importance of family-based interventions in managing childhood obesity and support the relevance of considering intergenerational transmission of health behaviors. This study particularly suggests that children’s health behaviors may be aligned with those of their legal guardians, with family members displaying similar behavioral profiles based on sex and WS, underscoring the important influence of legal guardians in shaping children’s lifestyle habits. The results also indicate that positive health perceptions and high levels of PA could be key factors in enhancing intervention success among children, supporting the need for personalized strategies that consider individual and family behavioral patterns. Overall, this study provides valuable insights into associations between a family and home-based intervention and health-related outcomes in the context of childhood obesity, while also emphasizing the added value of conducting family behavioral profiling in future interventions. Further research should explore the scalability of such approaches, including a better understanding of implementation aspects and the overall costs associated with delivering multi-professional, long-term family-based programs, in order to inform their potential replication in broader settings.

## Data Availability

Data supporting these findings are available from the corresponding author upon request. The data are not publicly available due to privacy or ethical restrictions.
