# Supplementary material for: Effects of a family home-based intervention on global health and 24h movements: an exploratory family cluster-based approach to childhood obesity
Source: Int J Public Health. 2026 Jun 25;71:1609165. doi: 10.3389/ijph.2026.1609165 (PMC13366504; doi:10.3389/ijph.2026.1609165)
Supplement: Supplementary file 1 [file Supplementaryfile1.docx]

**Supplementary File 1.** Design of the study (Auvergne, France, 2025)

CAPAS-Q: Children and Adolescents Physical Activity and Sedentarity Questionnaire, EQ5D-3L: 3-Levels European Quality of Life 5 Dimensions questionnaire, EQ5D-Y: 3-Levels European Quality of Life 5 Dimension for Youth, ONAPS PAQ: Physical Activity Questionnaire of the National Observatory on Physical Activity and Sedentarity, PEDSQL: Pediatric Quality of Life Inventory, SF-36: Short Form Questionnaire


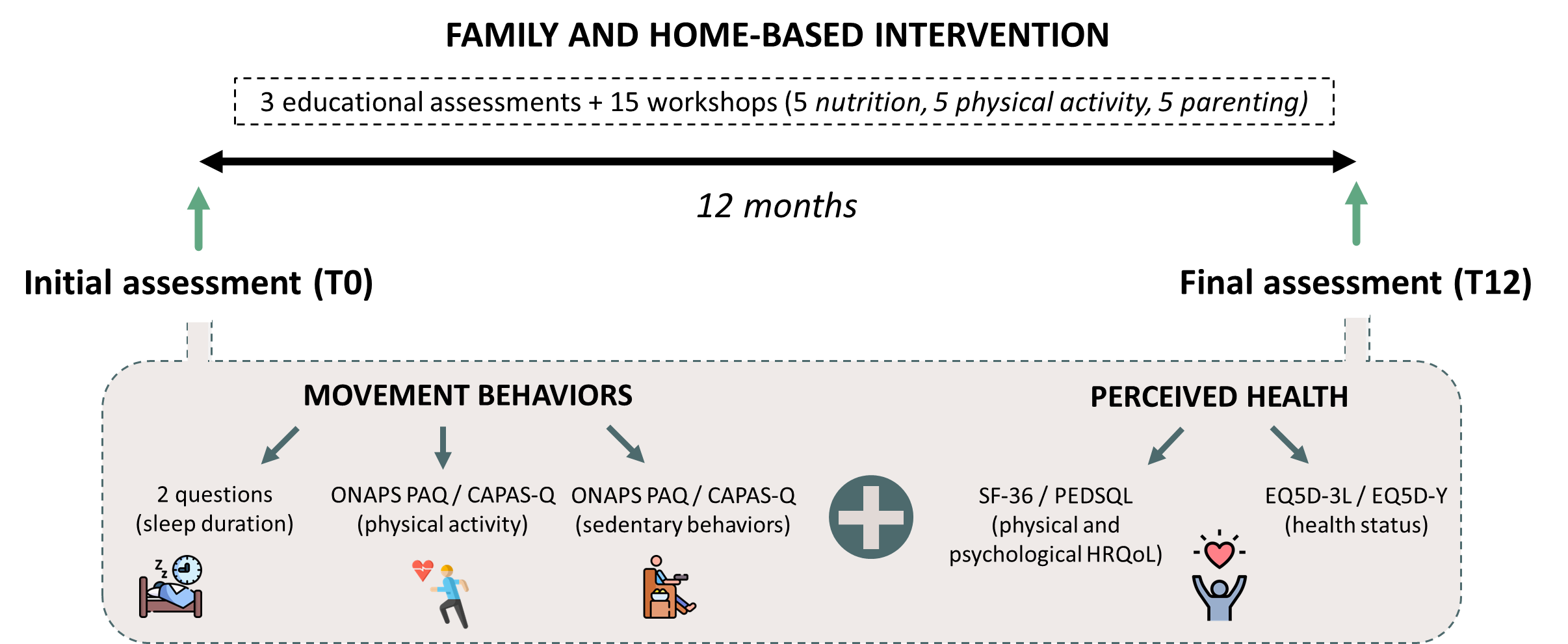


**Supplementary File 2.** Scoring method of questionnaires (Auvergne, France, 2025)

Abbreviations. CAPAS-Q: Children and Adolescents Physical Activity and Sedentarity Questionnaire, EQ5D-3L: 3-Levels European Quality of life 5 Dimensions questionnaire, HRQoL: Health-Related Quality of life, MPA: Moderate Physical Activity, ONAPS PAQ: Physical Activity Questionnaire of the National Observatory on Physical Activity and Sedentarity, PEDSQL: Pediatric Quality of Life Inventory, SF-36: Short Form Questionnaire, VPA: Vigorous Physical Activity

| **Legal guardians** | | | **Children** | | |
| --- | --- | --- | --- | --- | --- |
| *Questionnaire* | *Outcomes* | *Scoring calculation and interpretation* | *Questionnaire* | *Outcomes* | *Scoring calculation and interpretation* |
| **SF-36** | HRQoL : Physical and psychological health | Physical health = $\frac{\boldsymbol{q}\boldsymbol{1}\boldsymbol{+}\boldsymbol{2}\boldsymbol{+}\boldsymbol{3}\boldsymbol{a}\boldsymbol{,}\boldsymbol{b}\boldsymbol{,}\boldsymbol{c}\boldsymbol{,}\boldsymbol{d}\boldsymbol{,}\boldsymbol{e}\boldsymbol{,}\boldsymbol{f}\boldsymbol{,}\boldsymbol{g}\boldsymbol{,}\boldsymbol{h}\boldsymbol{,}\boldsymbol{i}\boldsymbol{,}\boldsymbol{j}\boldsymbol{+}\boldsymbol{4}\boldsymbol{a}\boldsymbol{,}\boldsymbol{b}\boldsymbol{,}\boldsymbol{c}\boldsymbol{,}\boldsymbol{d}\boldsymbol{+}\boldsymbol{7}\boldsymbol{+}\boldsymbol{8}\boldsymbol{+}\boldsymbol{11}\boldsymbol{a}\boldsymbol{,}\boldsymbol{b}\boldsymbol{,}\boldsymbol{c}\boldsymbol{,}\boldsymbol{d}}{\boldsymbol{22}}$  Psychological health =  $\frac{\boldsymbol{q}\boldsymbol{5 a,b,c+6+9}\boldsymbol{a,b,c,d,e,f, g,h, i+10}}{\boldsymbol{14}}$  Interpretation:  0-33: severe dependence  34-65: moderate dependence  66-100: independence | **PEDSQL** | HRQoL : Physical and psychological health | Physical health =  $\frac{\boldsymbol{sum of q 1\to8 'physical functionning'}}{\boldsymbol{8}}$  Psychological health =  $\frac{\boldsymbol{sum of q 1\to5'emotiona}\boldsymbol{l}^{\boldsymbol{'}}\boldsymbol{+'social, +'schoo}\boldsymbol{l}^{\boldsymbol{'}}\boldsymbol{functionning'}}{\boldsymbol{3}}$  Suggested Interpretation based on previous studies:  0-60: low quality of life  61-80: moderate quality of life  81-100: good quality of life |
| **EQ5D-3L** | Health status | Health status = $\frac{\boldsymbol{q}\boldsymbol{1}\boldsymbol{+}\boldsymbol{2}\boldsymbol{+}\boldsymbol{3}\boldsymbol{+}\boldsymbol{4}\boldsymbol{+}\boldsymbol{5}}{\boldsymbol{5}}$  Interpretation:  ≤ 1: very good  1 – 2: good  > 2: weak | **EQ5D-3L-Y** | Health status | Health status = $\frac{\boldsymbol{q}\boldsymbol{1}\boldsymbol{+}\boldsymbol{2}\boldsymbol{+}\boldsymbol{3}\boldsymbol{+}\boldsymbol{4}\boldsymbol{+}\boldsymbol{5}}{\boldsymbol{5}}$  Interpretation:  ≤ 1: very good  1 – 2: good  > 2: weak |
| **ONAPS PAQ** | Physical activity level and sedentarity time | Physical activity level = MPA_MET_ + VPA_MET_  MPA_MET_ = **4*[(q5*q6)+(q33*q34)] + 3.3*(q9*q10) + 6*(q12*q13)**  VPA_MET_ **= 8*[(q2*q3)+(q30*q31)]**  Interpretation:  < 600 METs.min^-1^.week^-1^: inactive  ≥ 600 METs.min^-1^.week^-1^ : active  Sedentarity time = $\frac{\boldsymbol{q}\boldsymbol{7+[}\left( \boldsymbol{q}\boldsymbol{18*q}\boldsymbol{19} \right)\boldsymbol{+}\left( \boldsymbol{q}\boldsymbol{24*q}\boldsymbol{25} \right)\boldsymbol{+(q}\boldsymbol{27*q}\boldsymbol{28)}}{\boldsymbol{7}}$  Interpretation:  < 3h.day^-1^: low sedentary time  3-7 h.day^-1^: moderate sedentary time  > 7h.day^-1^: high sedentary time | **CAPAS-Q** | Physical activity level and sedentary behaviors | Physical activity level = $\frac{\boldsymbol{q}\boldsymbol{1}\boldsymbol{\to}\boldsymbol{q}\boldsymbol{18}}{\boldsymbol{18}}$  Interpretation:  1 ≤ x ≤ 2 🡪 to improve  2 < x ≤ 3 🡪 it would be good to improve  x > 3 🡪 satisfactory, to be maintained  Sedentary behaviors = $\frac{\boldsymbol{q}\boldsymbol{19}\boldsymbol{\to}\boldsymbol{q}\boldsymbol{31}}{\boldsymbol{13}}$  Interpretation:  1 ≤ x ≤ 2 🡪 satisfactory, to be maintained  2 < x ≤ 4 🡪 it would be good to improve  x > 4 🡪 to improve |

**Supplementary File 3.** Descriptive characteristics of legal guardians and children involved in the ProxOb program (Auvergne, France, 2025)

Abbreviations. BMI: Body Mass Index, IOTF: International Obesity Task Force, NW: Normal Weight, OB: Obesity, OW: Overweight

^1^ n (% of the subsample). ^2^ Mean ± SD

|  | Female legal guardians  n=135 | Male legal guardians  n=88 | Children with OW/OB  n=194 | NW children  n=60 |
| --- | --- | --- | --- | --- |
| **Sex** ^1^ |  |  |  |  |
| *Females* | 135 (100) | 0 (0) | 102 (53) | 30 (50) |
| *Males* | 0 (0) | 88 (100) | 92 (47) | 30 (50) |
| **Age (years)** ² | 41.2 ± 6.7 | 44.7 ± 7.6 | 10.7 ± 3.1 | 10.6 ± 4.7 |
| **Weight (kg)** | 88.7 ± 24.2 | 101.2 ± 21.4 | 64.0 ± 23.9 | 42.0 ± 17.7 |
| **Height (cm)** | 163.6 ± 6.1 | 176.5 ± 6.7 | 149.1 ± 16.8 | 144.4 ± 25.2 |
| **BMI (kg/m²)** | 33.2 ± 9.0 | 32.4 ± 6.5 | 27.8 ± 6.4 | 18.9 ± 2.7 |
| **BMI z-score IOTF** | - | - | 2.5 ± 0.8 | 0.6 ± 0.7 |
| **Weight status** |  |  |  |  |
| *Normal weight* | 28 (21) | 13 (15) | 0 (0) | 60 (100) |
| *Overweight* | 28 (21) | 24 (27) | 72 (38) | 0 (0) |
| *Obesity* | 79 (58) | 51 (58) | 117 (62) | 0 (0) |
| **Family profile** |  |  |  |  |
| *Biparental* | 100 (74) | 82 (93) | 143 (74) | 41 (68) |
| *Single parental* | 35 (26) | 6 (7) | 51 (26) | 19 (32) |

**Supplementary File 4.** Baseline body mass index z-score, health-related quality of life, health status and movement behaviors between completers and non-completers children of the intervention (attrition analysis) (Auvergne, France, 2025)

Abbreviations. BMI: Body Mass Index, HRQoL: Health Related Quality of Life, IOTF: International Obesity Task Force.

^1^ n (% of the subsample). ^2^ Median [IQR], in accordance with the use of the non-parametric Kruskal-Wallis test

|  | Completers (n=34) | Non-completers (n=207) | p-value |
| --- | --- | --- | --- |
| **Sex ^1^** |  |  |  |
| *Females* | 21 (62) | 100 (48) | 0.275 |
| *Males* | 13 (38) | 107 (52) |  |
| **Age (years) ^2^** | 9.5 [7.0, 12.0] | 11.0 [9.0, 14.0] | **0.022** |
| **BMI (kg/m²)** | 23.6 [19.9, 28.2] | 25.4 [21.1, 29.3] | 0.282 |
| **BMI z-score IOTF** | 2.2 [1.4, 2.8] | 2.1 [1.4, 2.9] | 0.827 |
| **Weight status** |  |  |  |
| *Normal weight* | 6 (18) | 48 (24) | 0.677 |
| *Overweight* | 16 (47) | 97 (47) |  |
| *Obesity* | 12 (35) | 60 (29) |  |
| **Physical health (HRQoL)** | 84.4 [75.0, 87.5] | 81.3 [68.8, 90.6] | 0.486 |
| **Psychological health (HRQoL)** | 73.3 [63.3, 86.7] | 75.0 [63.3, 83.3] | 0.943 |
| **Health status** | 1.2 [1.2, 1.4] | 1.2 [1.0, 1.4] | 0.502 |
| **Sleep duration (hrs)** | 9.6 [9.4, 10.3] | 9.5 [9.4, 10.0] | 0.324 |
| **Physical activity level** | 1.9 [1.8, 2.3] | 2.2 [2.0, 2.5] | 0.255 |
| **Sedentary behaviors level** | 2.2 [1.8, 3.3] | 2.6 [2.2, 3.1] | 0.333 |

**Supplementary File 5.** Baseline body mass index, health-related quality of life, health status and movement behaviors between completers and non-completers guardians of the intervention (attrition analysis) (Auvergne, France, 2025)

Abbreviations. BMI: Body Mass Index, HRQoL: Health Related Quality of Life

^1^ n (% of the subsample). ^2^ Median [IQR], in accordance with the use of the non-parametric Kruskal-Wallis test

|  | Completers (n=53) | Non-completers (n=169) | p-value |
| --- | --- | --- | --- |
| **Sex ^1^** |  |  |  |
| *Females* | 35 (66) | 100 (59) | 0.348 |
| *Males* | 18 (34) | 70 (41) |  |
| **Age (years) ^2^** | 41.0 [39.0, 45.0] | 42.0 [38.0, 47.0] | 0.792 |
| **BMI (kg/m²)** | 31.9 [27.3, 36.6] | 31.65 [27.2, 36.6] | 0.828 |
| **Weight status** |  |  |  |
| *Normal weight* | 7 (13) | 34 (20) | 0.382 |
| *Overweight* | 35 (66) | 95 (56) |  |
| *Obesity* | 11 (21) | 41 (24) |  |
| **Physical health (HRQoL)** | 78.2 [62.0, 87.0] | 73.2 [56.4, 86.6] | 0.259 |
| **Psychological health (HRQoL)** | 60.7 [43.6, 75.7] | 61.4 [49.3, 75.0] | 0.721 |
| **Health status** | 1.4 [1.2, 1.4] | 1.4 [1.2, 1.6] | 0.946 |
| **Sleep duration (hrs)** | 7.8 [7.3, 8.3] | 8.0 [7.5, 8.8] | 0.284 |
| **Physical activity (METs.min^-1^.week^-1^)** | 21.9 [6.1, 87.8] | 39.5 [12.0, 145] | 0.177 |
| **Sedentary time (hrs)** | 6.1 [2.6, 9.1] | 7.6 [3.8, 10.3] | 0.113 |

**Supplementary File 6.** Identification of three clusters among children based on health-related quality of life and health status using principal component analysis : comparison of characteristics by cluster and distribution by sex and weight status (Auvergne, France, 2025)

Abbreviations. BMI: Body Mass Index, HRQoL: Health-Related Quality of life, OB: Obesity, OW: Overweight, Δ z-score BMI: z-score change from baseline (T0) to the end of the intervention (T12)

^1^ Mean ± SD [Range]

^2^ n (% in the cluster)

* p-value between T0 and T12 <0.05

|  | **Cluster 1 - Low global health (vulnerable profile)**  n=57 | **Cluster 2 – Moderate globale health (intermediate profile)**  n=65 | **Cluster 3 – High global health (favourable profile)**  n=26 |
| --- | --- | --- | --- |
| **Physical health (HRQoL)** ^1^ | 66.6 ± 9.6 [59.4, 75.0] | 87.2 ± 8.6 [81.3, 93.8] | 91.3 ± 8.5 [87.5, 100.0] |
| **Psychological health (HRQoL)** | 62.0 ± 12.0 [55.0, 70.0] | 80.2 ± 10.0 [73.3, 86.7] | 85.5 ± 9.5 [78.8, 90.0] |
| **Health status** | 1.5 ± 0.3 [1.4, 1.6] | 1.2 ± 0.2 [1.0, 1.2] | 1.1 ± 0.2 [1.0, 1.2] |
| **Sex** ^2^ |  |  |  |
| Females | 30 (53) | 37 (57) | 11 (42) |
| Males | 27 (47) | 28 (43) | 15 (58) |
| **Weight status** |  |  |  |
| Normal weight | 3 (5) | 0 (0) | 26 (100) |
| Overweight/Obesity | 54 (95) | 65 (100) | 0 (0) |
| **Δ z-score BMI children OW/OB** | -0.1 ± 0.3 [-0.9, 0.5] | -0.3 ± 0.4 [-1.4, 0.3] * | - |

**Supplementary File 7**. Identification of three clusters among children based on sleep duration, physical activity and sedentary behaviors using principal component analysis : comparison of characteristics by cluster and distribution by sex and weight status (Auvergne, France, 2025)

Abbreviations. BMI: Body Mass Index, hrs: hours, OB: Obesity, OW: Overweight, Δ BMI z-score: z-score change from baseline (T0) to the end of the intervention (T12)

^1^ Mean ± SD [Range]. ^2^ n (% in the cluster)

* p-value between T0 and T12 <0.05

|  | **Cluster 1 – Active girls with OW/OB**  n=30 | **Cluster 2 – Sedentary boys with OW/OB**  n=27 | **Cluster 3 – Moderately Active NW children**  n=15 |
| --- | --- | --- | --- |
| **Sleep durations (hrs)** ^1^ | 9.8 ± 1.0 [9.5, 10.4] | 9.5 ± 0.9 [9.0, 10.0] | 9.3 ± 0.5 [8.9, 9.6] |
| **Physical activity** | 2.0 ± 0.3 [1.8, 2.2] | 2.4 ± 0.4 [2.2, 2.6] | 2.2 ± 0.3 [2.0, 2.5] |
| **Sedentary behaviors** | 2.5 ± 0.7 [2.1, 2.9] | 2.7 ± 0.7 [2.2, 3.2] | 2.7 ± 0.7 [2.3, 3.1] |
| **Sex** ^2^ |  |  |  |
| Females | 30 (100) | 0 (0) | 5 (33) |
| Males | 0 (0) | 27 (100) | 10 (67) |
| **Weight status** |  |  |  |
| Normal weight | 0 (0) | 0 (0) | 15 (100) |
| Overweight/Obesity | 30 (100) | 27 (100) | 0 (0) |
| **Δ BMI z-score children OW/OB** | -0.3 ± 0.4 [-1.4, 0.5] * | 0.1 ± 0.5 [-0.4, 0.9] | - |

**Supplementary File 8.** Effect size of time (fixed-effects estimates, 95% confidence interval) on overall health and 24-hour movements in children (Auvergne, France, 2025).

The dots represent the estimated effect size and the vertical bars the 95% confidence interval. The dashed line indicates no effect (zero value). The variables in the left panel (body mass index z-score, health status, sleep duration, physical activity level, level of sedentary behavior) are expressed on a standardized scale (−1 to 1), while the variables in the right panel (physical health and psychological health) are expressed on the raw scale of the questionnaire (−20 to 20).

Abbreviations. BMI: Body Mass Index, HRQoL: Health-Related Quality of Life, IOTF: International Obesity Task Force

**Supplementary File 9.** Correlation between Δ body mass index z-score and Δ behaviors T0-T12 of children with overweight or obesity at baseline (Auvergne, France, 2025)

Abbreviations. BMI: Body Mass Index

|  | **Δ BMI z-score** | **p-value** | **sample size (n)** |
| --- | --- | --- | --- |
| Δ physical health | -0.4068 | 0.1324 | 20 |
| Δ psychological health | -0.1804 | 0.5199 | 20 |
| Δ health status | 0.1461 | 0.5893 | 22 |
| Δ sleep duration | -0.2000 | 0.6059 | 24 |
| Δ physical activity | -0.5000 | 0.6667 | 13 |
| Δ sedentary behaviors | -0.0286 | 0.9572 | 19 |

**Supplementary File 10.** Effect size of time (fixed-effects estimates, 95% confidence interval) on overall health and 24-hour movements in legal guardians (Auvergne, France, 2025). The dots represent the estimated effect size, and the vertical bars represent the 95% confidence interval. The dashed line indicates no effect (zero value). The variables are expressed on their respective raw scales: body mass index (kg/m²), physical and psychological health, and sedentary time (−20 to 20) ; health status and sleep duration (−1 to 1) ; and physical activity (−100 to 100).

Abbreviations. BMI: Body Mass Index, HRQoL: Health-Related Quality of Life,

**Supplementary File 11.** Changes in global health and movement behaviors outcomes between T0 and T12 among children with complete data : mixed-effects model results (sensitivity analysis) (Auvergne, France, 2025)

Abbreviations. BMI: Body Mass Index, HRQoL: Health Related Quality of Life, IOTF: International Obesity Task Force, WS: Weight Status

Values are presented as mean ± SD with significant results in bold.

A separate Wilcoxon test was performed. * p < 0.05, ** p<0.01, *** p<0.001.

|  | T0 | T12 | **Mixed Models effects** | | |
| --- | --- | --- | --- | --- | --- |
|  |  |  | Time | Weight status | Time x WS |
| **BMI z-score IOTF** | 2.2 ± 1.0 | 2.1 ± 1.0 | 0.056 | **<0.001** | **0.002** |
| **Physical health (HRQoL)** | 79.8 ± 12.8 | 82.7 ± 3.1 | 0.783 | 0.154 | 0.393 |
| **Psychological health (HRQoL)** | 71.0 ± 16.1 | 75.4 ± 12.4 | 0.604 | 0.142 | 0.287 |
| **Health status** | 1.4 ± 0.3 | 1.3 ± 0.2 | 1.000 | 0.155 | 0.420 |
| **Sleep duration (hrs)** | 9.7 ± 0.1 | 9.4 ± 1.0 * | 0.321 | 0.575 | 0.982 |
| **Physical activity level** | 2.1 ± 0.3 | 2.2 ± 0.4 | **0.050** | 0.743 | 0.096 |
| **Sedentary behaviors level** | 2.4 ± 0.5 | 2.8 ± 0.7 ** | 0.634 | 0.868 | 0.392 |

**Supplementary File 12.** Changes in global health and movement behaviors outcomes between T0 and T12 among legal guardians with complete data : mixed-effects model results (sensitivity analysis) (Auvergne, France, 2025)

Abbreviations. BMI: Body Mass Index, HRQoL: Health Related Quality of Life, WS: Weight Status

Values are presented as mean ± SD with significant results in bold.

A separate Wilcoxon test was performed. * p < 0.05, ** p<0.01, *** p<0.001.

|  | T0 | T12 | **Mixed Models effects** | | |
| --- | --- | --- | --- | --- | --- |
|  |  |  | Time | Weight status | Time x WS |
| **BMI (kg/m²)** | 32.3 ± 5.8 | 32.2 ± 5.5 | **0.036** | **<0.001** | **0.022** |
| **Physical health (HRQoL)** | 75.1 ± 13.8 | 80.3 ± 10.6 * | 0.251 | **0.002** | **0.004** |
| **Psychological health (HRQoL)** | 59.4 ± 18.7 | 64.0 ± 17.7 * | 0.829 | 0.438 | 0.237 |
| **Health status** | 1.4 ± 0.2 | 1.3 ± 0.3 * | 0.506 | 0.122 | 0.697 |
| **Sleep duration (hrs)** | 8.0 ± 0.7 | 8.1 ± 1.0 | 0.969 | 0.514 | 0.649 |
| **Physical activity (METs.min^-1^.week^-1^)** | 74.2 ± 106.3 | 89.6 ± 120.5 | 0.888 | 0.342 | 0.534 |
| **Sedentary time (hrs)** | 7.3 ± 3.6 | 7.9 ± 4.8 | 0.122 | 0.888 | 0.178 |

**Supplementary File 13.** Detailed sample size for each outcome in each subgroup and at both T0 and T12 time points (Auvergne, France, 2025)

Abbreviations. BMI: Body Mass Index, HRQoL: Health-Related Quality of life, IOTF: International Obesity Task Force, NW: Normal Weight, OB: Obesity, OW: Overweight, T0: baseline, T12: after 1-year intervention

|  | **All children** | | **NW children** | | **Children with OW/OB** | | **All legal guardians** | | **Female legal guardians** | | | | **Male legal guardians** | | | |
| --- | --- | --- | --- | --- | --- | --- | --- | --- | --- | --- | --- | --- | --- | --- | --- | --- |
|  | T0  n=254 | T12  n=73 | T0  n=60 | T12  n=16 | T0  n=194 | T12  n=57 | T0  n=223 | T12  n=99 | T0  n=135 | | T12  n=68 | | T0  n=88 | | T12  n=31 | |
|  |  |  |  |  |  |  |  |  | NW  n=28 | OW/OB  n=107 | NW  n=11 | OW/OB  n=57 | NW  n=13 | OW/OB  n=75 | NW  n=4 | OW/OB  n=27 |
| **BMI z-score IOTF** | 241 | 34 | 54 | 6 | 187 | 28 | - | - | - | - | - | - | - | - | - | - |
| **BMI (kg/m²)** | - | - | - | - | - | - | 223 | 53 | 28 | 107 | 11 | 57 | 13 | 75 | 4 | 27 |
| **Physical health (HRQoL)** | 163 | 37 | 32 | 7 | 131 | 30 | 154 | 71 | 18 | 83 | 8 | 39 | 7 | 46 | 3 | 21 |
| **Psychological health (HRQoL)** | 159 | 37 | 30 | 6 | 129 | 31 | 156 | 87 | 17 | 78 | 10 | 50 | 9 | 52 | 3 | 24 |
| **Health status** | 158 | 39 | 30 | 7 | 128 | 32 | 166 | 90 | 18 | 85 | 9 | 53 | 9 | 54 | 4 | 24 |
| **Sleep duration** | 113 | 39 | 24 | 8 | 89 | 31 | 131 | 70 | 15 | 65 | 8 | 42 | 9 | 42 | 2 | 18 |
| **Physical activity** | 80 | 28 | 18 | 7 | 62 | 21 | 108 | 57 | 12 | 53 | 8 | 31 | 5 | 38 | 2 | 16 |
| **Sedentary time/behaviors** | 103 | 35 | 19 | 8 | 84 | 27 | 110 | 59 | 14 | 55 | 8 | 35 | 7 | 34 | 2 | 14 |
